# European experience of steroid therapy in children with developmental and epileptic encephalopathy with spike wave activation in sleep ((D)EE-SWAS)

**DOI:** 10.1186/s13023-025-03725-0

**Published:** 2025-04-29

**Authors:** Dilan Canbay, Floor E. Jansen, Jan Schönberger, Victoria San Antonio-Arce, Julia Jacobs, Kerstin Alexandra Klotz

**Affiliations:** 1 Department of Neuropediatrics and Muscle Disorders, Faculty of Medicine, Albert-Ludwig-University Freiburg, Breisacherstr. 62, 79106, Member of the European Reference Network (ERN) EpiCARE, Freiburg, Germany; 2 Department of Pediatric Neurology, Brain Center University Medical Center Utrecht, Heidelberglaan 100, 3584 AE, Member of the European Reference Network (ERN) EpiCARE, Utrecht, The Netherlands; 3 Freiburg Epilepsy Center, Medical Center, Faculty of Medicine, University of Freiburg, University of Freiburg, Breisacherstr. 64, 79106, Member of the European Reference Network (ERN) EpiCARE, Freiburg, Germany; 4 Hotchkiss Brain institute and Alberta Children’s Hospital Research Institute, University of Calgary, 2500 university drive NW, T2N1N4, Calgary Alberta, Calgary, Canada; 5 Department of Neuropediatrics, Universital Hospital Bonn, Venusberg-Campus 1, 53127, Member of the European Reference Network (ERN) EpiCARE, Bonn, Germany

**Keywords:** Epilepsy, Steroids, ESES, CSWS, SWAS, Cognition

## Abstract

**Background:**

Developmental and epileptic encephalopathy with spike wave activation in sleep (DEE-SWAS) and epileptic encephalopathy with spike wave activation in sleep (EE-SWAS) are rare but well-known childhood epileptic disorders. Steroids are one of the first line treatment options, but a variety of steroid regimens exists. The aim of this survey was to evaluate the practices of steroid therapy in the treatment of (D)EE-SWAS across European centers.

**Methods:**

An online survey was conducted (via ‘SurveyMonkey’ Europe) among European epilepsy centers. Questions asked included: characteristics of replying center, applied definition of DEE-SWAS, existence of regional/national guidelines regarding diagnostic and therapeutic management. Particular attention was paid to the indication/contraindication of steroids and treatment regimens used.

**Results:**

Responses were obtained from 60 centers across 18 countries. Only 15% of centers reported the availability of national guidelines for the management of (D)EE-SWAS. There were variations in definition of (D)EE-SWAS, with Spike-Wave- Index (SWI) > 85 (irrespective of cognitive status) and SWI > 50% with concurrent neurodevelopmental regression being the most prevalent, reported in 36% and 50%, respectively. Steroids and clobazam were considered the predominant treatment options, with the primary indication for steroids being neurodevelopmental arrest (52%) and failure of clobazam treatment (51%). Treatment goals of steroid treatment primarily focused on neurodevelopmental improvement (95%), and reduction of SWI (66%). Methylprednisolone and prednisone were the most frequently used steroids, although other steroid types were also reported. Pulse therapy was utilized exclusively in 47% of centers. The most commonly used steroid regimen was intravenous/oral methylprednisolone pulse therapy (20 mg/kg/day for 3 days, either monthly or weekly), although a broad variety of different regimens were reported. Criteria influencing decisions about steroid treatment were largely based on personal experience, with scientific publications playing a role in decision-making in only 14% of centers.

**Conclusion:**

Steroids are part of the first line therapy of (D)EE-SWAS across Europe, but heterogeneity in formulations, dosages, and regimens persists due to limited guideline availability. The absence of comparative studies and the discordant definitions of (D)EE-SWAS further hinder comparisons of treatment efficacy. We recommend that harmonizing steroid treatment strategies is imperative for optimizing (D)EE-SWAS management.

**Supplementary Information:**

The online version contains supplementary material available at 10.1186/s13023-025-03725-0.

## Background

Developmental and epileptic encephalopathy with spike wave activation in sleep ((D)EE-SWAS) is characterized by a neurocognitive regression or plateauing associated with an EEG pattern of marked increase of epileptiform activity in transition from wakefulness to nonREM sleep [1]. Initially, (D)EE-SWAS was defined by the requirement of epileptic activity during at least 85% of non-rapid eye movement (NREM) sleep-EEG; however, cases with a spike-wave index (SWI) of 50–85% have since been incorporated into the spectrum and the recent definition does not specify a request a particular threshold [1–3].

Furthermore, alongside the debate surrounding the SWI-threshold, there existed ambiguity in terminology prior to the most recent ILAE classification of epilepsy syndromes. Some authors employed the term Electrical Status Epilepticus in Sleep (ESES) to denote the EEG pattern characterized by frequent spike waves in sleep, while referring to the clinical manifestation of encephalopathy with sleep-induced epileptic discharges as Continuous Spikes and Waves during Slow Wave Sleep (CSWS). Conversely, other authors utilized the terms ESES and CSWS interchangeably [4–6]. With the introduction of the new classification of epilepsy syndromes, the syndromic presentation of children with the EEG-pattern of ESES and the clinical picture of an encephalopathy with neurodevelopmental regression or arrest was more precisely defined. Two terms have been employed to delineate the clinical spectrum of these conditions: Epileptic Encephalopathy with Slow Wave Activation in Sleep (EE-SWAS) in patients with previously normal development and Developmental and Epileptic Encephalopathy with Slow Wave Activation in Sleep (DEE-SWAS) observed in patients with pre-existing neurodevelopmental disorders [1]. These conditions manifest with various combinations of cognitive, language, behavioral, and motor regression, typically occurring within weeks from the onset of the EEG pattern. Seizures may be present, but the most burdensome symptom of the disease is usually neurodevelopmental decline. Although the EEG pattern of (D)EE-SWAS often resolves during puberty, cognitive deficits frequently persist. For brevity, we will use the term “(D)EE-SWAS” to encompass both DEE-SWAS and EE-SWAS throughout this paper.

Early and appropriate treatment of (D)EE-SWAS is considered crucial to prevent further cognitive decline or even improve cognitive functions. Various conventional antiseizure medications (ASMs), benzodiazepines, steroids, intravenous immunoglobulins, ketogenic diet, and, in selected cases, epilepsy surgery have been utilized for (D)EE-SWAS treatment [7, 8]. Treatment decisions have historically been guided by expert opinions, leading to wide variations in treatment strategies among clinicians [6]. A meta-analysis in 2015 suggested the superior efficacy of steroids in (D)EE-SWAS [2]. The recently published RESCUE ESES trial was ended prematurely due to feasibility reasons but nevertheless showed a difference in efficacy favoring steroids as well [9]. However, despite steroids being recognized as a first-line treatment option, a range of different steroid regimens have been published and no comparative data on efficacy or tolerance are available [10]. The objective of this project was to assess the standards and practices of steroid therapy for treating ESES in various centers across Europe. For the purpose of this study, we used the term ESES to refer to both the EEG-pattern and the associated disorder with encephalopathy but will refer to the re-named term (D)EE-SWAS in this paper.

## Methods

We developed a structured and stratified questionnaire (additional file 1) utilizing an online survey tool (SurveyMonkey, Portland, OR, USA) and invited specialized centers treating children and adolescents with epilepsy across eighteen European countries to participate. The identification of potential participants was facilitated through regional subchapters of the International League Against Epilepsy (ILAE), trough the European Reference Network (ERN) EpicCare and the official websites of national epilepsy organizations, utilizing direct outreach or mailing lists. Within each center, only one physician was contacted and requested to respond on behalf of the entire department or to forward the invitation to a colleague within the same center. In the survey introduction, participants were notified that, for the study’s purposes, no distinction was drawn between the terms (D)EE-SWAS, ESES and CSWS.

The survey commenced with inquiries about the practitioner’s professional qualifications and experience in treating patients with (D)EE-SWAS. Subsequent questions addressed general aspects of (D)EE-SWAS, including the availability of national guidelines for its treatment, the existence of standardized departmental treatment protocols (SOPs) for patients with (D)EE-SWAS, and the applied definition of (D)EE-SWAS in the respondent’s center. Additional inquiries focused on the management, treatment, and monitoring of patients with (D)EE-SWAS. In the second segment, the survey explored steroid treatment specifics, covering indications, contraindications, treatment goals, and the utilization of SOPs for steroid treatment in (D)EE-SWAS. The questionnaire also investigated the type of steroid employed, the nature of treatment (pulsatile or continuous), applied steroid regimens, and the rationale behind the chosen steroid type and regimen. Descriptive statistical analysis was conducted using GraphPad Prism (V. 10.2.0, GraphPad Software, San Diego, CA, USA), presenting categorical variables as absolute numbers and percentages, and quantitative data as mean and standard deviation or median and range. Percentages correspond to the number of responses for each respective question.

## Results

Of the 102 centers invited to participate, 60 responses from 18 European countries were included in the analysis. More than half of the countries (*n* = 10) were represented by a single center or maximum of two centers (Table 1). The majority of respondents were pediatric neurologists (*n* = 42, 70%). Among responders, 68% were working in a university hospital, 13% at an epilepsy center, and 10% at a neuropediatric or neurology department of a non-university hospital. Most responders indicated that their center manages between 2 and 5 patients with ESES per year (47%, *n* = 28). Additionally, 20% reported caring for 11–20 patients annually, 15% for 6–10 patients, 10% for 1 patient, and 8% for more than 20 patients annually.

**Table 1:** Number of responding centers from each country

Country	Responders *n* (%)
Germany	22 (36.6)
Italy	7 (11.6)
France	6 (10)
Switzerland	3 (5)
Croatia	3 (5)
Austria	3 (5)
Portugal	3 (5)
Spain	2 (3.3)
Hungary	2 (3.3)
Belgium	1 (1.7)
Denmark	1 (1.7)
Finland	1 (1.7)
Latvia	1 (1.7)
Lithuania	1 (1.7)
Romany	1 (1.7)
Scottland	1 (1.7)
Sweden	1 (1.7)
United Kingdom	1 (1.7)

Only 15% reported the availability of national guidelines for diagnostic and treatment, while 5% were not certain. Curiously, among the responders from Germany (*n* = 22), 4 confirmed the presence of national guidelines, 17 denied and 1 was uncertain. Similarly, in France, half of the responders confirmed, and half denied the availability of national guidelines. SOPs were reported to be available in 45% of all centers. Respondents were queried about the typical definition of (D)EE-SWAS employed in their respective centers. Table 2 illustrates the various definitions utilized.

**Table 2:** Different definitions applied (multiple answers possible)

Definition applied	Responders *n* (%)
SWI > 50% *associated with* neurodevelopmental delay, arrest or regression	30 (50.0)
SWI > 85% *regardless* of cognition	22 (36.7)
SWI > 85% *associated with* neurodevelopmental delay, arrest or regression	11 (18.3)
Strong activation of SW (no threshold) *associated with* neurodevelopmental arrest or regression	5 (8.3)
SWI > 50% *regardless* of cognition	2 (3.3)
Other*	1 (1.7)

SW = spike-waves; SWI = spike-wave-index

*Other: SWI > 60%

**Fig. 1:**
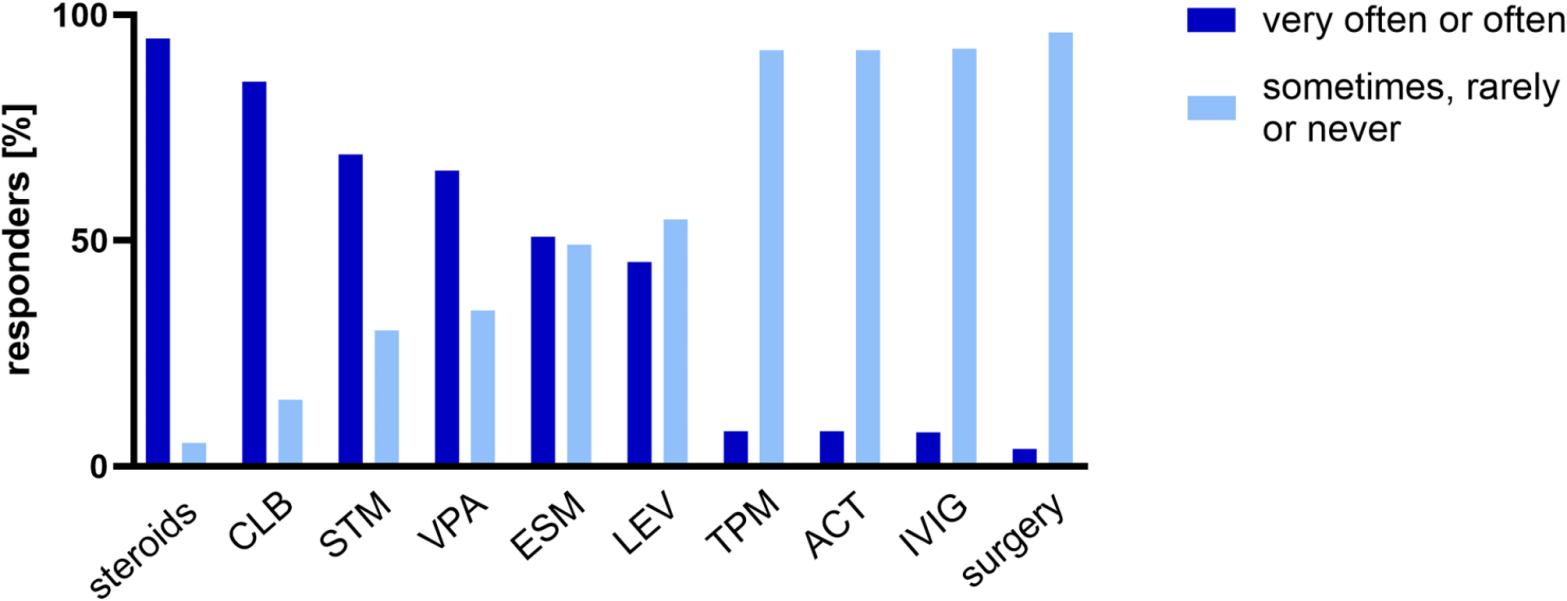
Percentage of responders who reported the frequency of prescription for various therapies, categorized as very often or often versus sometimes, rarely, or never. CLB = clobazam; STM = sulthiame; VPA = valproate; ESM = ethosuximide; LEV = levetiracetam; TPM = topiramate; ACT = acetazolamide; IVIG = intravenous immunoglobulins; surgery = epilepsy surgery

The treatments most frequently prescribed were steroids, clobazam, sulthiame and valproate (Fig. 1). Of 58 responders, *n* = 33 would treat patients with (D)EE-SWAS and identified structural or genetic etiology differently than patients with (D)EE-SWAS of unknown etiology, with considerations such as epilepsy surgery in cases with unilateral structural etiology, avoidance of steroids as a first-line treatment in cases with structural or genetic etiology and using ethosuximide as a first-line treatment in cases without know etiology. The majority of responders treated patients with (D)EE-SWAS mainly as outpatients (60.3%). Reasons for admission included steroid treatment or diagnostic procedures, among others.

Responders were asked about monitoring practices in children with (D)EE-SWAS. Routine EEG was always or usually performed by 67% of responders, sleep EEG (daytime nap) by 79%, video EEG including whole-night recording by 64%, neurodevelopmental testing by 86%, and psychiatric evaluation by 68%. SOPs specifically for steroid treatment in (D)EE-SWAS were reported to be available in 59%. In instances where no SOP was accessible, steroid treatment followed a somewhat standardized regimen that would be individually adapted for each patient (52%). Alternatively, individual decision-making without a standardized regimen was reported by 30%, while only 17% adhered to the same regimen consistently.

Indications for steroid treatment included neurodevelopmental arrest/regression (52%), failure of clobazam treatment (51%), failure of any other ASMs (46%), or clear activation of spike waves in sleep without necessity of neurodevelopmental deficits (27%). Most responders stated to apply a SWI threshold for indication of steroid treatment. Specifically, 32% of responders indicated that steroid treatment would not be considered if the SWI was less than 85%, while 12% stated this threshold as less than 50%, irrespective of other symptoms. Reported treatment goals included neurodevelopmental improvement (95%), improvement of behavioral problems or psychiatric comorbidities (68%), reduction of SWI (66%), improvement of sleep architecture (53%), reduction of seizures (29%), and achieving seizure freedom (22%). Responders were queried about the line of steroid treatment in (D)EE-SWAS, and most stated they could not provide a definitive answer, as the decision was taken on an individual basis for each patient (36%). Among them, 24% considered steroids always as a second-line therapy, and 19% would only use them as a first line therapy in case of neurodevelopmental arrest or regression. Only 10% would consider steroids always as a first-line therapy, and 7% would contemplate using steroids not earlier than as a third-line treatment. The most common reasons to discontinue steroid therapy were improvement in neurodevelopmental function (89%), overall lack of improvement (67%) after a self-specified time range (reported time median 6 months (range 1–12)), and adverse events (58%).

**Fig. 2:**
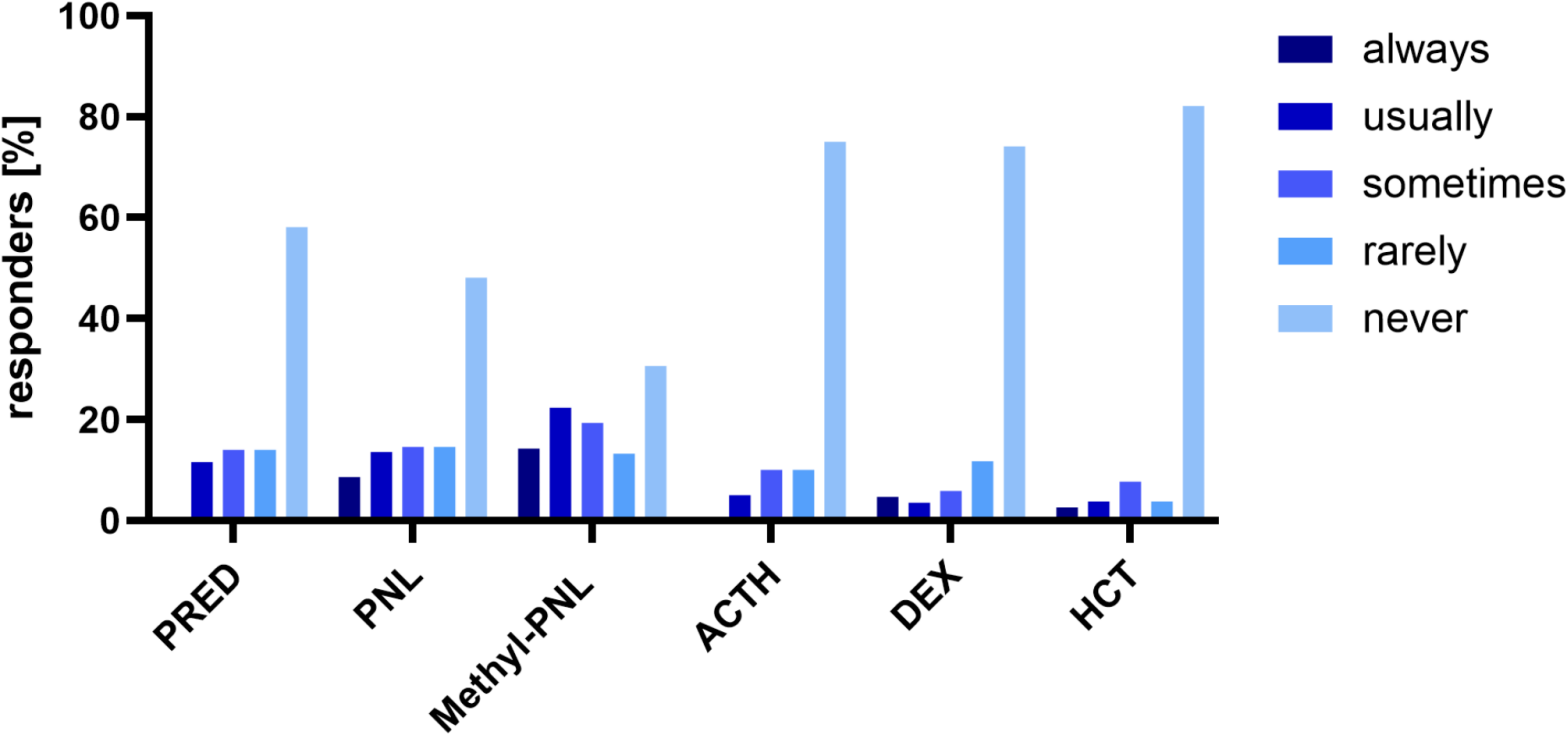
Percentage of responders who reported the frequency of utilization for various steroids, categorized as always, usually, sometimes, rarely or never. PRED = prednisone; PNL = prednisolone; Methyl-PNL = methylprednisolone; ACTH = adrenocorticotropic hormone; DEX = dexamethasone; HCT = hydrocortisone

Participants in the survey were questioned about the frequency of using specific steroids in the clinical management of (D)EE-SWAS, selecting responses from “always” to “never.” Despite “never” being the predominant response across all steroids, it is noteworthy that each steroid type was nevertheless reported to be in use, with minor distinctions observed between responses indicating “usually,” “sometimes,” and “rarely.” Methylprednisolone exhibited the smallest differentials among all these categories (Fig. 2). Most respondents (47%) exclusively utilized pulse therapy, with an additional 23% employing a combination of pulse followed by continuous therapy. Only 16% primarily opted for continuous therapy, and 14% indicated an individualized approach in their steroid regimen decisions. These treatment choices were largely based on personal experience and only in a minority (14%) based on scientific publications. Best tolerance (55%), best long-term efficacy (45%), and best short-term efficacy (34%) were reasons named for the selection of the above-mentioned therapy regimens. The most common relative contraindications stated were hypertension (87%), a history of cardiac disease (79%), diabetes mellitus (68%), and obesity (52%), whereas only 16% regarded diabetes mellitus as an absolute contraindication.

Monitoring practices during pulse/continuous steroid therapy were comprehensive, with the majority of respondents monitoring always/often blood pressure (98/100%), weight (93/98%), blood glucose (64/88%), urine glucose (62/73%), and echocardiograms (62/66%). Regarding the duration of steroid treatment, the responses varied, with 42% opting for at least one month, another 42% continuing treatment for at least six months, and only 9% stated to continue steroid treatment until neurodevelopmental improvement was observed. Discontinuation of steroid therapy was predominantly prompted by improvements in neurodevelopment (89%), followed by an overall lack of improvement after a median of 6 months (range 1 to 12 months) for 67% of respondents, and adverse events for 58%.

**Fig. 3:**
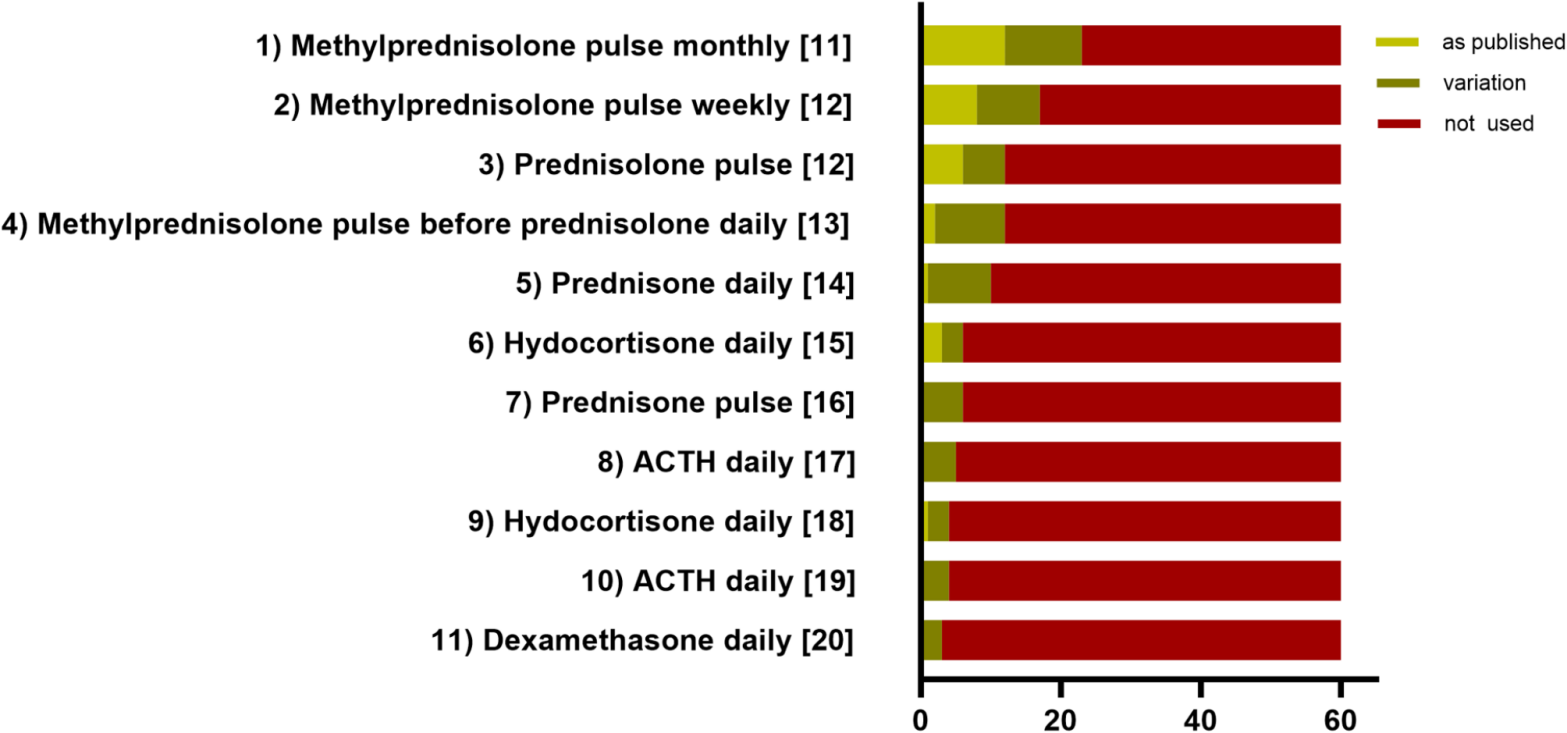
Number of respondents who reported the application for various steroid regimens, either as published or in variation or not at all. (1) Methylprednisolone intravenously (iv) 20 mg/kg/d 3 days per month for 6 months [11]; (2) Methylprednisolone iv or orally 20 mg/kg/d 3 days per week for 4 weeks, continue individually [12]; (3) Prednisolone orally 20 mg/kg/d 3 days per week for ≥ 4 weeks modified from [12]; (4) Methylprednisolone iv daily starting from 30 mg/kg/d weaning over 10 days, followed by prednisolone orally 2 mg/kg/d daily for another 6 months [13]; (5) Prednisone orally 1 mg/kg/d daily for 6 months [14]; (6) Hydrocortisone orally 5 mg/kg/d daily, decreasing dosage in monthly steps for several months [15]; (7) Prednisone orally 2 mg/kg/d weekly for 6 weeks [16]; (8) Synthetic ACTH intramuscularly (im) 0.01-0.04 mg/kg/d daily for 11–43 days [17]; (9) Hydrocortisone orally 20 mg/kg/d daily for up to several months [18]; (10) ACTH im 0.01-1 mg/kg/d daily in repetitive courses of for 6–15 days [19]; 11) Dexamethasone orally 0.15 mg/kg/d daily for up to several weeks [20]

At the end of the survey, eleven different published steroid regimens were listed, along with reference to an exemplary paper where one of the listed regimens was utilized. These regimens encompassed various steroid types, modes of administration, dosages, durations, as well as pulse or continuous therapy. Respondents were then asked if they implemented the regimens exactly, similarly, or not at all. It was reported that only 7 out of the 12 regimens were used exactly, although all regimens were reported to be applied similarly by at least one responder (Fig. 3). Of the 60 responders, 27 (45%) used at least one regimen exactly with 24 (40%) of them exclusively using one regimen precisely. Furthermore, respondents had the option to indicate the use of another published or personally developed regimen that they regularly employed. Five responders reported utilizing dexamethasone 20 mg/m²/d for 3 days followed by pulses every 1–4 weeks, while one responder stated the use of methylprednisolone 20-30 mg/kg for 5 days without report of further continuation of steroid treatment. Notably, no obvious associations were observed between the regimens used and the country of residence of the responder.

## Discussion

(Developmental) and Epileptic Encephalopathy with spike wave activation in sleep represents a rare condition characterized by a high burden of disease, particularly acquired neurodevelopmental deficits. Recent evidence suggests that early steroid treatment may have a positive impact on neurodevelopmental outcome [9]. However, despite this emerging evidence, there is currently a lack of established guidelines or best practice recommendations for steroid treatments in (D)EE-SWAS patients. Moreover, a wide array of steroid regimens has been published in smaller cohorts. Nevertheless, comparative studies are not available. We performed a survey to provide an overview of the opinions and practices of steroid treatment in (D)EE-SWAS applied at European centers.

The diverse definition of (D)EE-SWAS used in the past presents the first difficulty in aligning observational metrics and therapeutic strategies. Our survey identified variations in definitions, with the SWI > 85% (irrespective of neurodevelopmental status) and SWI > 50% with concurrent neurodevelopmental regression being the most commonly used singular definitions. The heterogeneous use of terminology for the condition and its identifying characteristics is in line with a previous survey among members of the Child Neurology Society and the American Epilepsy Society and has also been recognized as a major obstacle in a recent review [5, 21]. Furthermore, the quantification of epileptiform activity during sleep lacks standardization, leading to diverse methodologies in selecting EEG samples, and determine SWI [22]. Interestingly, in the free-text comments of the survey, several respondents remarked that establishing thresholds for defining epileptiform activity is inherently arbitrary. Meanwhile, the ILAE has released new definitions of epilepsy syndromes occurring in childhood. The definitions of EE-SWAS and DEE-SWAS do not set a specific threshold but emphasize the pronounced activation of abnormalities during sleep and their temporal associations with cognitive, behavioral, or motor regression [1]. However, implementing teaching courses on the new diagnostic criteria, for example within the European network of EpiCare, could help standardize their application. Adhering strictly to these criteria may enhance comparison between centers in future research endeavors.

Responders indicated a lack of national guidelines for (D)EE-SWAS, despite conflicting responses. Nonetheless, at least half of the centers reported having SOPs, highlighting some level of organizational standardization. However, individual decision-making still holds significant importance, as only 17% of responders reported consistently adhering to the same steroid regimen. This aligns with the findings of a previous survey about (D)EE-SWAS treatment practices, which also revealed diverse treatment approaches among experts in North America [6].

In our survey, the primary indication for steroid therapy in (D)EE-SWAS was neurodevelopmental decline, aligning with the primary treatment goal of neurodevelopmental improvement (95%), closely followed by the secondary indication of clobazam non-response. Consequently, the most common reason for discontinuing steroid therapy was improvement of neurodevelopment. Interestingly, in the previously mentioned survey among physicians in North America, the most commonly considered outcome measure of treatment efficacy was epileptiform EEG activity, prioritized above improvement of neurodevelopment [6]. The pathophysiological mechanisms underlying encephalopathy in connection with sleep-related activation of epileptic discharges remain incompletely understood. However, available data support the synaptic homeostasis hypothesis explaining that the excessive activation of epileptic discharges during sleep disrupts the downscaling of number and strength of synapses, a process that typically occurs during physiological sleep [23]. Therefore, the observed shift in treatment goals between the two surveys does not necessarily indicate a change in approach but rather a refinement in defining clinically relevant outcome measures.

Numerous steroid regimens have been published; however, the absence of randomized controlled trials for most steroid types and regimens, as well as the lack of head-to-head comparative studies, renders assessment of efficacy impossible. Our survey indicates that various published regimens are applied to some extent, albeit in somewhat modified versions. The most commonly reported regimen was methylprednisolone pulse therapy with a dosage of 20 mg/kg/day for 3 days every four weeks [11], closely followed by the same regimen but with weekly intervals instead of monthly [12]. In contrast, the survey by Sánchez-Fernandez in 2014 indicated a preference for daily oral prednisone therapy, whereas only 15% of respondents in our survey opted for daily administration of prednisone as a first-line therapy over pulse therapy [6]. This divergence may reflect regional differences or changes over time. Notably, the treatment choices, including the type, line, and regimen of steroid therapy, were reported to be largely based on personal experience in our survey, with only a minority (14%) relying on scientific publications.

It is essential to consider the limitations of our survey when interpreting the findings. First, the questionnaire primarily comprised closed-ended questions, enabling efficient collection of quantitative data but potentially limiting the depth of information gathered. Overall, the responder rate of 60% is considered satisfactory for this type of survey. However, the survey’s lack of representativeness is noteworthy, with respondent distribution varying among European countries. Similar distributions have been observed in other European survey studies [24]. German responders were dominantly prevalent, which may be attributed to the authors’ background as well as the decentralized nature of care in Germany. Furthermore, there is a likelihood of overrepresentation of respondents from larger centers, potentially leading to the underrepresentation of physicians and centers with less experience. Despite the instructions provided on how to define the term (D)EE-SWAS for this survey, the potential for different interpretations among authors and ambiguity in terminology and concepts may have influenced the results. It’s important to note that not all available regimens might have been represented; however, the primary goal of the survey was to illustrate trends rather than encompass every treatment possibility.

## Conclusions

In conclusion, our survey reveals significant variability in the preparations, doses, and steroid regimens applied in (D)EE-SWAS. However, the limited quality of available data and inconsistent definition of (D)EE-SWAS pose challenges for direct efficacy comparison. Prospective comparable trials in the near future seem unlikely, given experiences with recruitment difficulties in the RESCUE ESES trial. Nonetheless, our findings underscore the urgent need for aligning steroid treatment recommendations. Expert consensus could serve as a compromise solution in this endeavor, and ERN EpiCARE could support these efforts towards harmonizing clinical practices in rare and complex epilepsies.

## Electronic supplementary material

Below is the link to the electronic supplementary material.

**Supplementary Material 1**: **Additional file 1**: Original survey

## Data Availability

The datasets used and analyzed during the current study are available from the corresponding author on reasonable request.
