# Supplementary material for: European experience of steroid therapy in children with developmental and epileptic encephalopathy with spike wave activation in sleep ((D)EE-SWAS)
Source: Orphanet J Rare Dis. 2025 Apr 29;20:204. doi: 10.1186/s13023-025-03725-0 (PMC12039249; doi:10.1186/s13023-025-03725-0)
Supplement: Supplementary file 1 — Supplementary Material 1: Additional file 1: Original survey [file 13023_2025_3725_MOESM1_ESM.pdf]

ESES

Dear collegues,

Thank you for your consideration to participate in this comprehensive survey on steroid treatment in children with Electrical Status Epilepticus in Sleep (ESES) in Europe.

Replying the survey takes approximately 5 minutes. We are aiming for an overview of common practices per center, so we kindly ask you to answer the survey only once per center.

For the purpose of this study, we do not differ between the terms "ESES" and "CSWS" (continuous spike waves during sleep). Spike wave index during non-REM sleep is abbreviated as SWI.

During the active phase of the survey, data protection policies are strictly observed, IP addresses are not recorded. Data storage and evaluation is only done in an anonymous matter. Correlation of data to participants or to a center is not possible.

For further information please contact us at any time. We appreciate your time and consideration for contribution.

Kind regards,

Dr. Alexandra Klotz and Prof. Dr. Julia Jacobs (Freiburg/Germany) and Dr. Floor Jansen (Utrecht/Netherlands)

contact für this survey:
Department of Neuropediatrics and Muscle Disorders
University of Freiburg- Medical Center
Mathildenstr. 1; 79106 Freiburg
Phone.: 0761-270-43000; Fax.: 0761-270-43500
Email: kerstin.alexandra.klotz@uniklinik-freiburg.de
https://www.uniklinik-freiburg.de/kinderklinik/behandlungsspektrum/epilepsiezentrum.html

ESES

Part 1 General information

1. Please select all statements that apply for your person.
I am.....

- ◯ ... a general pediatrician
- ◯ ... a neuropediatrician
- ◯ ... a neurologist
- ◯ ... formally trained as a epileptologist
- ◯ ... head of the center
- ◯ ... a consultant in this center
- ◯ ... an epilepsy (research) nurse in this center
- ◯ ... other, please specify

[ ]

2. I am working at ...

- ◯ ... a Neuropediatric/Neurology department of a university hospital
- ◯ ... Neuropediatric/Neurology department of a non-university hospital
- ◯ ... an Epilepsy Center
- ◯ ... other (please specify)

[ ]

3. In which country is your Center located?

- ◯ Austria
- ◯ Belgium
- ◯ Croatia
- ◯ Czechoslowakia
- ◯ Denmark
- ◯ Estonia
- ◯ Finland
- ◯ France
- ◯ Georgia
- ◯ Germany
- ◯ Greek
- ◯ Hungary
- ◯ Ireland
- ◯ Italy
- ◯ Latvia
- ◯ Lithuania
- ◯ Netherlands
- ◯ Norway
- ◯ Poland
- ◯ Portugal
- ◯ Romania
- ◯ Scottland
- ◯ Spain
- ◯ Sweden
- ◯ Switzerland
- ◯ UK
- ◯ ... other (please specify)

[ ]

4. How many patients with ESES (as per definition used in your center) are seen at your center per year?

- ◯ 1 or less
- ◯ 2-5
- ◯ 6-10
- ◯ 11-20
- ◯ >20

ESES

Part 2 - General aspects of ESES

5. Are there national guidelines for diagnostic criteria and treatment of ESES in your country?

- ◯ Yes
- ◯ No
- ◯ I am not sure

6. Does your center have a standardized departmental treatment protocol (SOP) for patients with ESES?

- ◯ Yes
- ◯ No

7. What definition(s) of ESES is/are used at your center? (SWI=spike wave index during non-REM sleep, all definitions mean bilateral SWI)

- ☐ SWI >85% REGARDLESS of cognition
- ☐ SWI >85% PLUS cognitive delay, arrest or regression
- ☐ SWI >50% REGARDLESS of cognition
- ☐ SWI >50% PLUS cognitive delay, arrest or regression
- ☐ Strong activation of interictal epileptiform activity in non-REM sleep without SWI threshold
- ☐ Other (please specify)

8. Please rate how often the following treatment options are prescribed for ESES at your center

	very often	often	sometimes	rarely or never
Sultiame	◯	◯	◯	◯
Clobazam or other benzodiazepines	◯	◯	◯	◯
Steroids	◯	◯	◯	◯
Valproate	◯	◯	◯	◯
Ethosuximide	◯	◯	◯	◯
Acetazolamid	◯	◯	◯	◯
Ketogenic diet	◯	◯	◯	◯
Levetiracetam	◯	◯	◯	◯
Topiramate	◯	◯	◯	◯
Intravenous immunglobulins	◯	◯	◯	◯
Epilepsy surgery	◯	◯	◯	◯

ESES

9. Is treatment at your Center different for "idiopathic" (including atypical rolandic epilepsy and Landau Kleffner syndrome) versus "symptomatic" ESES?

- ○ Yes
- ○ No
- ○ Please comment shortly what is different for symptomatic ESES

10. How are ESES-patients managed at your center? Please select all that apply.

- ☐ Always as outpatients
- ☐ Always as inpatients
- ☐ Mainly as outpatients, inhouse only for diagnostics
- ☐ Mainly as outpatients, inhouse only for steroid treatment

11. Please rate if ESES-patients at your center are repeatedly (at least 2 times) monitored for

	always	usually	sometimes	rarely	never
Routine EEG	○	○	○	○	○
Sleep EEG (up to 2 hrs)	○	○	○	○	○
Overnight sleep EEG or video-EEG	○	○	○	○	○
Neurocognitive testing	○	○	○	○	○
Behavioral or psychiatric problems (consultation of psychiatrist or questionnaires)	○	○	○	○	○

ESES

Part 3 specific questions about steroids for ESES treatment

* 12. Does your center have a standardized departmental treatment protocol (SOP) for steroid treatment in ESES (or a section about steroids in the general ESES-protocol)?

- ◯ Yes
- ◯ No

ESES

13. How is steroid treatment performed in general at your Center?

- ◯ Steroid treatment is based on individual desicion and varies from case to case
- ◯ Steroid treatment is basicly based on one typical scheme that will be adaped for individual patients
- ◯ Even without a formal SOP, steroid tretment always follows the same scheme

14. What are the indications of steroids in ESES-patients at your center? (please select all that apply)

- ☐ Just ESES diagnosis (regardles of SWI and neurocognitive impairment)
- ☐ Only if SWI > 85% uni- or bilaterally
- ☐ Only if SWI >50% bilaterally or >85% unilaterally
- ☐ As soon as neurocognitive arrest is present
- ☐ Only if neurocognitive regression is present
- ☐ Only if seizures are present
- ☐ Failure of Clobazam
- ☐ Failure of any other antiseizure medication

15. What are the main treatment goals for steroids in ESES patients at your center?

- ☐ Reduction of SWI
- ☐ Improvement of sleep architecture in EEG
- ☐ Reduction of seizure frequency >50%
- ☐ Seizure freedom
- ☐ Neurocognitive improvement
- ☐ Improvement of behavioral problems and/or psychiatric comorbidities as ADHS

ESES

16. Steroid treatment for ESES at your center is considered as ...

- ◯ ... always first line
- ◯ ... first line only if SWI is >85% bilaterally
- ◯ ... first line only if neurocognitive regression or arrest are present
- ◯ ... always second line
- ◯ ... not earlier than third line
- ◯ ... only in case of secondary relapse
- ◯ I can't answer the question, first or second line depends too much on individual constellations

17. Please rate how often the following steroids are used at your center for ESES

	always	usually	sometimes	rarely	never
Prednison orally	◯	◯	◯	◯	◯
Prednison intravenously	◯	◯	◯	◯	◯
Prednisolon orally	◯	◯	◯	◯	◯
Prednisolon intravenously	◯	◯	◯	◯	◯
Methylprednisolon orally	◯	◯	◯	◯	◯
Methylprednisolon intravenously	◯	◯	◯	◯	◯
ACTH intramuscularly	◯	◯	◯	◯	◯
Dexamethason orally	◯	◯	◯	◯	◯
Dexamethason intraveously	◯	◯	◯	◯	◯
Hydrocortisone orally	◯	◯	◯	◯	◯
Hydrocortisone intravenously	◯	◯	◯	◯	◯

ESES

18. What is the preferred type of steroid therapy at your center

- ◯ Pulse therapy only
- ◯ Continuous therapy only
- ◯ Pulses first followed by daily doses
- ◯ I can't answer the question because it is an individual decision

19. What are the reasons for the above mentioned steroid type and frequency? Please select all that apply.

- ☐ Best short-term efficacy based on own experience
- ☐ Best long-term efficacy (prevention of relapses) based on own experiences
- ☐ Best tolerance (lowest adverse events) based on own experience
- ☐ Availability of different steroids
- ☐ National guidelines
- ☐ Scientific publication (if possible please enter author +/- year below)
- ☐ Not applicable, we use several different types of steroids

Publication:

20. Please state if steroid treatment is regarded as contraindicated at your center if one of the following comorbidities is present

	absolute contraindication	relative contraindication	no contraindication
Obesity	◯	◯	◯
Diabetes melitus	◯	◯	◯
Metabolic disorder	◯	◯	◯
High blood pressure	◯	◯	◯
History of cardiac disorder	◯	◯	◯

ESES

21. How often are the following check-ups performed in case of steroid PULSE treatment? (either at your center or recommended by you to be done by general pediatrician)

	always	usually	sometimes	rarely	never
Blood pressure	○	○	○	○	○
Urine glucose	○	○	○	○	○
Blood glucose	○	○	○	○	○
Echocardiography	○	○	○	○	○
Body measurements	○	○	○	○	○

22. How often are the following check-ups performed in case of CONTINUOUS steroid treatment? (either at your center or recommended by you to be done by general pediatrician)

	always	usually	sometimes	rarely	never
Blood pressure	○	○	○	○	○
Urine glucose	○	○	○	○	○
Blood glucose	○	○	○	○	○
Echocardiography	○	○	○	○	○
Body measurements	○	○	○	○	○

23. How long is steroid treatment for ESES usually performed at your center (good tolerance provided)? Please select all that apply.

- ☐ At least one month (pulses or continuous treatment)
- ☐ At least 6 months (pulses or continuous treatment)
- ☐ At least until SWI <50% bilaterally
- ☐ At least until neurocognitive improvement is seen
- ☐ None of the above

24. What would be the reasons to end steroid therapy for ESES at your center? Please select all that apply.

- [ ] Predefined time per protocol or standards
- [ ] Occurence of arterial hypertension
- [ ] Fasting blood glucose > 200mg/dl
- [ ] Weight gain resulting in obesity
- [ ] other (moderate to severe) adverse events
- [ ] SWI < 50%
- [ ] Normalization/remarkable improvement of language and or cognitive function
- [ ] No improvement of SWI and/or cognitive functioning after .... (please enter time below)
- [ ] Time

____

ESES

25. Please look at the following steriod schemes that have been used in previous studies and select if one is similar to the one used at your center.

	exactly the same	similar	not used
**Prednisone** 2mg/kg weekly for 6 weeks *(Hempel et al 2019)*	○	○	○
**Prednisolone** 20mg/kg on 3 days per week, duration 4 weeks, then re-evaluation *(modified Bast et al 2014)*	○	○	○
**Prednisone** 1mg/kg daily for 6 months *(Sinclair & Snyder 2006)*	○	○	○
**ACTH** 0.01-1mg/kg/d daily in repetitive courses of for 6-15 days *(Altunel et al 2017)*	○	○	○
**ACTH** 0.01-0.04mg/kg daily for 11-43 days *(Inutsuka et al 2006)*	○	○	○
**Dexamethasone** 0.15mg/kg daily for 4 weeks, slowly decreasing after *(Chen et al 2016)*	○	○	○
**Methylprednisolone** 20mg/kg on 3 days every 4 weeks, duration 6 months *(Munckhof 2016)*	○	○	○
**Methylprednisolone** 20mg/kg on 3 days per week, duration 4 weeks, then re-evaluation *(Bast et als 2014)*	○	○	○
**Methylprednisolone** 30mg/kg daily than weaning, followed by Prednisolon 2mg/kg daily for 6 months *(Okuzay et al 2005)*	○	○	○
**Hydocortisone** 5mg/kg daily for 1 month, then reducing over 9 months total *(Buzatu 2009)*	○	○	○
**Hydocortisone** 20mg/kg daily for	○	○	○

several months
(Verhelst et al 2005)

Please decribe shortly "your" scheme if not listed above

ESES

End of survey

We thank you very much for participating in our survey and appreciate your time!
If there are any questions please do not hesitate to contact us at any time
kerstin.alexandra.klotz@uniklinik-freiburg.de
